# Gonadotropin-Releasing Hormone Analogue Treatment in Females with Moderately Early Puberty: No Effect on Final Height

**DOI:** 10.4274/jcrpe.2356

**Published:** 2016-06-06

**Authors:** Şenay Savaş-Erdeve, Zeynep Şıklar, Bülent Hacıhamdioğlu, Pınar Kocaay, Emine Çamtosun, Gönül Öcal, Merih Berberoğlu

**Affiliations:** 1 Ankara University Faculty of Medicine, Department of Pediatrics, Division of Pediatric Endocrinology, Ankara, Turkey

**Keywords:** early puberty, gonadotropin-releasing hormone analog, final height

## Abstract

**Objective:** To investigate the effects of treatment with gonadotropin-releasing hormone analog (GnRHa) on final height in girls who experienced moderately early puberty with symptoms beginning at 7-8.5 years of age.

**Methods:** Female cases diagnosed with moderately early puberty which had started between ages 7 to 8.5 years were included in the study. In the treatment groups, all cases with a bone age ≤10.5 years constituted group 1 (n=18) and those with a bone age >10.5 years constituted group 2 (n=23). The 8 patients for which treatment approval could not be obtained constituted group 3. The 49 cases in all three groups were observed until they reached their final height.

**Results:** Target height, target height standard deviation score (SDS), final height, and final height SDS values were similar in all 3 groups. Final height showed a significant positive correlation with target height (p=0.000, r=0.54) and height at diagnosis (p=0.003, r=0.467) in all groups. Linear regression analysis revealed that a 1 cm longer height at diagnosis increased the final height 0.213 fold, and a 1 cm longer target height at diagnosis increased the final height 0.459 fold.

**Conclusion:** We found that GnRHa did not make a positive contribution to final height in cases of moderately early puberty.

## WHAT IS ALREADY KNOWN ON THIS TOPIC?

There are only a few studies on the effects of gonadotropin-releasing hormone analog (GnRHa) treatment on final height in cases with borderline early puberty.

## WHAT THIS STUDY ADDS?

GnRHa therapy did not make a positive contribution to final height in cases aged 7-8.5 years with early puberty in this study.

## INTRODUCTION

Early puberty is defined as puberty that begins at an earlier age than the age accepted as “normal”. Moderately early puberty is not a rare condition (1). There is no single age range which defines moderately early puberty; in girls, studies have reported age ranges of 8-10 years (2,3), 7.5-8.5 years (4), 8-9 years (5), and 6-8 years (6).

Moderately early puberty is a paraphysiological condition, in reference to the earlier appearance of pubertal signs (4). Such children can reach Tanner stage 4 before 10 years of age, rather than the normal age of 11.9±1 years. This may lead to psychosocial problems and has also been reported to stunt growth (2,4,7,8). Data on the effectiveness of treatment in this group are very limited. Most relevant studies were not randomized and did not include a control group (9).

We investigated the effects of treatment with gonadotropin-releasing hormone analog (GnRHa) on final height in girls who experienced early puberty with symptoms beginning at 7-8.5 years of age, and determined whether the group that received early treatment, when their bone age (BA) was younger, showed a better effect regarding final height than the group that received treatment when bones were more mature. We followed the participants until they reached their final height.

## METHODS

Female cases who presented to the Pediatric Endocrinology Outpatient Clinic with symptoms of puberty starting at 7-8.5 years of age were included in the study. The Tanner and Marshall criteria were used for puberty staging. Patients who had at least Tanner stage 2 breast development were assessed as cases of early puberty (10). Height and body weight measurements were taken in the morning using a SECA® 767 height and weight meter (Carson City, NV, USA). Body mass index (BMI) was calculated as kg/m2. Height and BMI were compared to standard curves for Turkish children. Height standard deviation score (SDS) and BMI SDS were calculated according to calendar age. Cases with a BMI SDS >2 were considered obese and those with a BMI SDS of 1-2 were considered overweight (11,12).

A left hand wrist graph was obtained from all cases and BA was determined according to the Greulich and Pyle method (13). The predicted final height (PFH) based on BA was calculated according to the Bayley-Pinneau method (14). PFH SDS was determined for each case. Height achieved when the epiphyses were closed and when the growth rate within the last 1 year was <1 cm were considered to represent final height (15). Maternal and paternal heights were measured at the outpatient follow-ups, and target height (TH) and SDS were determined (16).

Luteinizing hormone (LH), follicle-stimulating hormone (FSH), and 17β-estradiol (E2) were measured using a morning blood sample in all cases. A basal serum LH level ≥0.3 IU/L (as long as it was consistent with the findings) was accepted as activation of the hypothalamic-pituitary-gonadal (HPG) axis (17). Cases with a basal LH level <0.3 IU/L underwent the standard stimulation test of 100 µg GnRH (Ferring Pharmaceuticals Inc., Parsippany, NJ, USA) with an intravenous injection between 8.00 and 8.30 AM to assess the patient for early puberty, and blood samples were taken at 0, 40, 60, 90, and 120 min to measure serum LH and FSH levels. Peak LH ≥5 IU/L was accepted as the diagnostic criterion for activation of the HPG axis (17).

Cranial magnetic resonance imaging was performed to exclude an organic lesion in cases diagnosed with early puberty using basal or peak LH level at the time of the GnRH stimulation test (18). Cases with such lesions were excluded from the study. Cases with problems that could affect growth and puberty, such as growth hormone deficiency, thyroid pathology, adrenal and gonadal pathology, dysmorphic syndrome, skeletal dysplasia, chronic illness, learning disability, cerebral palsy, hydrocephalus, and those with a history of chronic drug use were also excluded.

All cases underwent pelvic ultrasonography at the time of diagnosis to evaluate the consistency of the pubertal findings with the pelvic ultrasonography findings. Uterus length >3.5 cm, ovary volume >1.5 cm3, and a visible endometrial echo were accepted as criteria supportive of early puberty (19).

Forty-nine cases were diagnosed with idiopathic central early puberty (CEP). A family information booklet was provided to all families. The 41 patients whose families consented to the treatment schedule were administered a standard 3.75 mg dose of depot leuprolide acetate subcutaneously once every 28 days. These 41 patients were divided into two groups based on BA. All cases with a BA ≤10.5 years constituted group 1 (n=18) and those with a BA >10.5 years constituted group 2 (n=23). Eight patients for whom treatment approval could not be obtained constituted group 3 (control).

The patients were evaluated as followed in our outpatient clinic at 3-month intervals during treatment. Their compliance with the treatment and pubertal findings were recorded on observation forms. Morning height and body weight measurements were taken at each evaluation. BMI, height SDS, and BMI SDS were calculated. A hormonal evaluation was also performed once every 3 months. The cases underwent a standard intravenous GnRH stimulation test within 3-4 days after administration of the third dose of depot leuprolide. Peak LH <3 IU/L on this test was accepted as a suppressed HPG axis (20). The 3.75 mg depot leuprolide injections were continued once every 28 days in suppressed cases. Follow-up was continued with LH measurements in blood samples taken before the GnRHa injection and at minute 120 after the GnRHa injection at 3-month intervals. A peak LH response <4 IU/L on the GnRH analogue test was accepted as a criterion for a suppressed HPG axis (21) and the treatment was stopped in these cases. Regressing breast development and achieving prepubertal basal and GnRH- or GnRHa-stimulated LH levels were also accepted as HPG axis suppression criteria and a cause for cessation of treatment. HPG axis suppression was ensured in all treated cases.

BA was evaluated annually during treatment. The bone maturation ratio was calculated as the ΔBA/Δ chronological age (ΔBA/ΔCA) ratio using the annual change in the years following treatment compared to that at treatment initiation. PFH and PFH SDS at the start of the treatment and during years 1 and 2 of treatment were used to evaluate the short-term effectiveness of the treatment on height.

Treatment was terminated based on an individual evaluation of each patient at the earliest CA of 10 years after considering Tanner stage at the start of the treatment, BA, growth rate during the last course of treatment, and the request of the child and the family. The follow-up continued after terminating treatment until cases reached their final height. Final height and final height SDS were determined.

Gonadotropin levels in serum were measured with an Access DXI 800 (Beckman Coulter, Brea, CA, USA) device using the immune chemiluminescence method in all cases. The detection limit for LH and FSH was 0.2 mIU/mL. Serum E2 levels were measured with the Modular E170 Immunological analyzer system (Roche Diagnostics, Manheim, Germany) using the electro-chemiluminescence method. The detection limit for E2 was 5 pg/mL.

All data were evaluated using the Statistical Package for the Social Sciences 15.0 statistics program (SPSS, Inc., Chicago, IL, USA) at our biostatistics department. The chi-square test was used to compare percentage values between the groups. Mean values were compared using the t-test when the distribution was normal, the Mann-Whitney U-test when the data were not normally distributed and when comparing just two groups, and analysis of variance (ANOVA) or the Kruskal-Wallis test when the three groups were compared. The intra-group time comparisons were made with the paired t-test when the distribution was normal, the Wilcoxon test when the data were not normally distributed, and repeated-measures ANOVA and the Friedman’s analysis when the number of times was more than two. A p-value <0.05 was considered significant for all tests. Values are presented as means ± two standard deviations or median (range). Spearman’s rho correlation was used for non-parametric correlation statistical analyses.

## RESULTS

Mean age at diagnosis of the 49 female cases with early puberty was 8.65±0.81 years and the median pubertal stage was Tanner 3. The mean symptom duration was 11 months (1-72 months). Puberty was reported to have started with premature pubarche in three cases (6.1%). Only 3 of the 49 cases had a history of preterm birth. Mean birth weight was 3100 g. Retardation of intrauterine growth was present in three cases (6.1%), and all three were term births. Mean age of diagnosis of the total group was 8.65±0.81 years, pubertal stage 3±0.81, height SDS 1.39±0.92, BMI SDS 1.02±0.91, BA 10.5±1.5 years, BA/CA 1.12±0.12, THSDS -0.22±0.94, predicted height standard deviation score (PHSDS) -0.11±1.00. Mean age of diagnosis was 8.09±0.42 years, pubertal stage 3±0.57, height SDS 0.96±0.73, BMI SDS 0.81±0.81, BA 9.42±0.87, BA/CA 1.15±0.08, THSDS -0.26±0.83, PHSDS 0.08±0.92 in group 1, while mean age of diagnosis in group 2 was 9.03±0.74 years, pubertal stage 4±0.73, height SDS 1.78±0.83, BMI SDS 1.28±0.86, BA 11.63±0.78, BA/CA 1.28±0.09, THSDS -0.4±0.9, PHSDS -0.45±1.08. In group 3, mean age of diagnosis was 8.83±1.04 years, pubertal stage 2±1.03, height SDS 1.22±1.18, BMI SDS 0.79±0.72, BA 9.98±2.05, BA/CA 1.1±0.12, THSDS 0.38±1.12, PHSDS 0.39±0.59 (Table 1).

The demographic data and anthropometric characteristics of the study groups at the time of diagnosis are compared in Table 2. Duration of symptoms, maternal menarche age, maternal height, paternal height, TH, and BMI SDS were similar in the three groups. Age, pubertal stage, height SDS, BA, and BA/CA were significantly higher in group 2 compared to group 1 at diagnosis. The BA/CA ratio was similar in groups 1 and 3 and it was significantly higher in group 2 compared to group 3.

The first and second year follow-up data of the treatment groups are compared in Table 3. Height SDS, BMI SDS, growth velocity (GV), and GV SDS at the first and second year follow-up were similar in groups 1 and 2, while ΔBA/ΔCA in group 2 during the first and second years of follow-up was significantly lower than that in group 1 (p=0.009).

PFH and SDS at the time of diagnosis, PFH and SDS during the first year of follow-up, final height, final height SDS, TH, and TH SDSs were similar among the three groups (Table 4). PFH and SDS at the second year follow-up were lower in group 1 than in group 2 (p=0.021).

**Figure 1:**
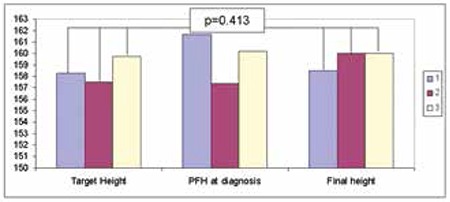
Target height, predicted final height at diagnosis and final height comparison

Final heights were similar among the groups (p=0.403) (Figure 1) and were positively correlated with TH (p=0.000, r=0.54) and height at the time of diagnosis (p=0.003, r=0.467). A linear regression analysis showed that for each 1 cm of height at the time of diagnosis, the final height increased by 0.213 times and for each 1 cm of TH, the final height increased by 0.459 times.

## DISCUSSION

Whether there is a height benefit of GnRHa administration when early puberty is diagnosed at 6-8 years of age is a controversial issue. Very few studies have been conducted with GnRHa in cases with advanced puberty starting at 8-10 years of age (8,22,23). The lack of control groups in these studies has raised unanswered questions regarding the effectiveness of treatment in these age groups (2). We compared the effects of GnRHa treatment on final height in early puberty cases whose symptoms started at 7-8.5 years of age with a control group and found that GnRHa did not make a positive contribution to final height. The group with advanced BA was taller at the time of diagnosis and their increase in BA slowed with treatment, leading to no regression in final height with treatment. Administering GnRHa to cases according to BA did not improve final height; final height in this age group was positively influenced only by TH and height at the time of diagnosis.

Lazar et al (24) observed that the rate of growth decreased after treatment in cases with early puberty diagnosed after 6 years of age and interpreted this as a negative effect of the intrinsic changes at the growth plate before the treatment. However, Brito et al (25) did not find a significant relationship between the CA at the start of treatment and linear growth after treatment. Although age at the start of treatment was negatively associated with final adult height in that study, GnRHa treatment was started after the age of 6 years in most girls who reached a normal final height. This led to the conclusion that genetic TH potential in girls >6 years can be preserved with GnRHa treatment. Similarly, Carel et al (26) reported a significant increase in adult height and predicted adult final height with treatment in 42 patients when puberty started at 6-8 years of age.

The final height of 75% of the cases was consistent with TH in a meta-analysis in which more than 637 female cases treated with GnRH analogues were evaluated (27). When final height was compared to PFH the start of treatment, the best results were obtained in patients treated earlier. However, no positive effect was found in PFH after GnRHa treatment of girls whose puberty started at 8-10 years of age. Cassio et al (4) reported results similar to ours in which mean final height was similar in their groups and not different than the TH in their study; they treated 23 of 46 patients whose puberty started at 7.5-8.5 years of age and followed the other half without treatment. Final height was equal to or greater than TH in 14 of the 20 patients who reached their final height in the treated group and in 12 of 18 patients who reached their final height in the untreated group. They reported that BA more advanced than the CA and a height age/BA ratio <0.9 could be prognostic criteria for a poor initial height prognosis. The final height of these patients was significantly lower than that of patients with a good initial height prognosis; however, treating cases with a poor initial height prognosis did not contribute to final height. The authors reported that treatment had no positive effect on final height in cases of moderately early puberty and that administering treatment to cases with a good or poor prognosis according to the height prognosis did not contribute to final height. The authors concluded that final height is only affected by height at the beginning of puberty and TH, and that height prognosis before treatment is not corrected by treatment. Our results are very similar to these findings. However, Cassio et al (4) emphasized that their data may have been influenced by the number of patients with a poor starting height prognosis being included in the study. We found a lower BA progression rate with treatment in the group with more advanced BA compared to the group with a younger BA. The similar results in the groups with and without advanced BA indicate that the treatment does not have an effect on final height independent of BA in this age group. Magiakou et al (28) similarly reported that final height in cases with idiopathic CEP starting at age of 7.9 years and treated with GnRHa was not different than that in the untreated group. Tanaka et al (29) monitored children with CEP in two prospective clinical studies (phase 2 and phase 3 studies) where they studied the effects of leuprorelin acetate on CEP. They were able to follow 76 (63 girls and 13 males) of these children until they reached their final height. These authors reported that 90% of the female cases with a CA of 7.7±2.2 years and a BA of 10.2±1.5 years at the start of the treatment achieved a final height in the TH range.

We found that GnRHa administration did not contribute to final height in cases aged 7-8.5 years with early puberty. Height at the time of diagnosis in the group with advanced BA was greater and BA progression was slower with treatment, leading to no change in final height with treatment. Administering GnRHa to cases according to BA did not improve final height, and the final height in this age group was positively influenced only by TH and height at the time of diagnosis. In our study, the number of girls in the control group was low and this state has caused a weakness of the study power.

In conclusion, factors such as age at menarche and the psychological condition of the child should be considered rather than height when deciding whether to start GnRHa treatment in girls showing signs of moderately early puberty.

**Ethics**

Ethics Committee Approval: It was taken from Ankara University Faculty of Medicine, Informed Consent: It was taken.

Peer-review: External peer-reviewed.

## AUTHORSHIP CONTRIBUTIONS

Surgical and Medical Practices: Şenay Savaş Erdeve, Merih Berberoğlu, Concept: Şenay Savaş Erdeve, Merih Berberoğlu, Gönül Öcal, Design: Şenay Savaş Erdeve, Merih Berberoğlu, Data Collection or Processing: Şenay Savaş Erdeve, Zeynep Şıklar, Bülent Hacıhamdioğlu, Pınar Kocaay, Emine Çamtosun, Merih Berberoğlu, Gönül Öcal, Analysis or Interpretation: Şenay Savaş Erdeve, Merih Berberoğlu, Literature Search: Şenay Savaş Erdeve, Writing: Şenay Savaş Erdeve, Merih Berberoğlu.

Financial Disclosure: The authors declared that this study received no financial support.

## Figures and Tables

**Table 1:**
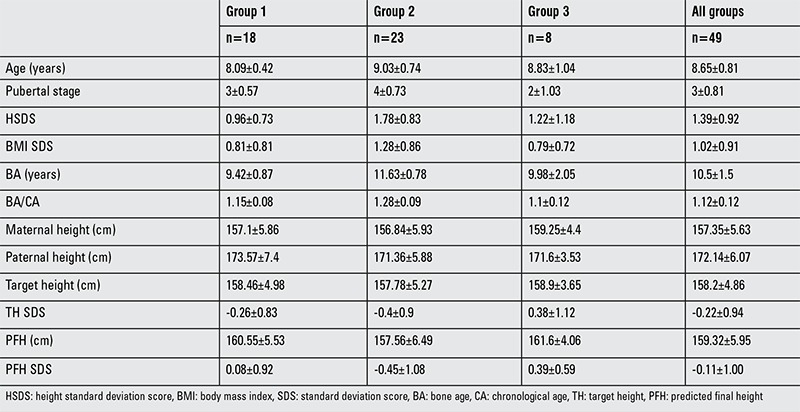
Characteristics of the three groups at the time of diagnosis

**Table 2:**
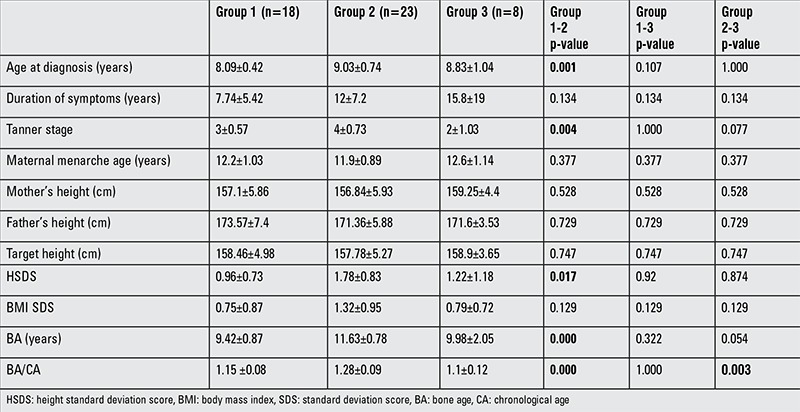
Comparison of demographic characteristics and anthropometric data of the study groups at the time of diagnosis

**Table 3:**
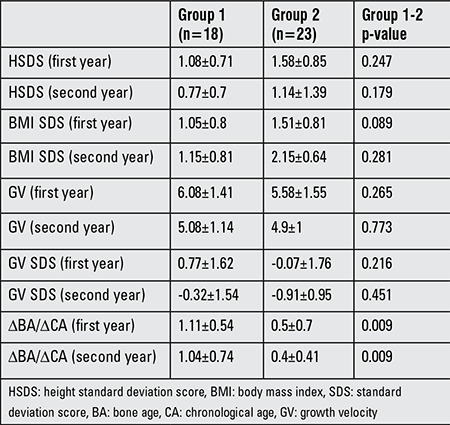
Comparison of the 1st and 2nd year follow-up values of the study groups

**Table 4:**
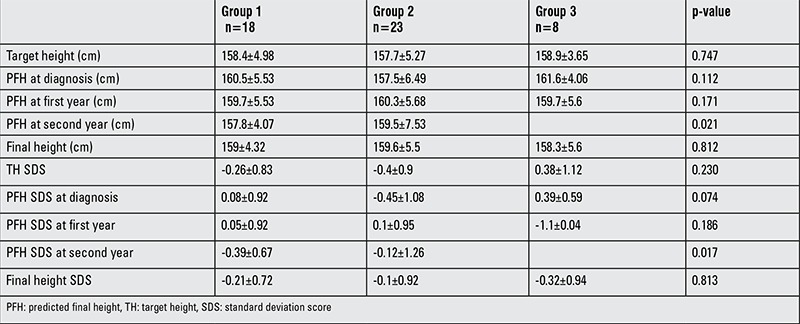
Comparison of target height, predicted final height, final height, and the standard deviation score values of these parameters between the groups
